# Effect of High-Pressure Treatments on the Properties of Food Packaging Materials with or without Antimicrobials

**DOI:** 10.3390/polym14245535

**Published:** 2022-12-17

**Authors:** Belén Soriano Cuadrado, Antonio Peñas Sanjuan, Javier Rodríguez López, Irene Delgado Blanca, Maria José Grande, Rosario Lucas, Antonio Galvez, Rubén Pérez Pulido

**Affiliations:** 1 Andaltec Plastic Technological Center, 23600 Martos, Spain; 2 Department of Health Sciences, University of Jaen, 23071 Jaén, Spain

**Keywords:** packaging materials, high hydrostatic pressure, antimicrobials

## Abstract

The aim of this research work was the comparative study of the different properties of interest in the case of plastic materials for food use before and after being subjected to treatment by high hydrostatic pressure (HHP) as well as the impact of additivation with antimicrobials. This method of food preservation is currently on the rise and is of great interest because it is possible to extend the shelf life of many foods without the need for the use of additives or thermal processing, as is the case with other preservation methods currently used. The effects of HHP treatment (680 MPa for 8 min) on plastic materials commonly used in the food industry were studied. These materials, in sheet or film form, were polyethylene (PE), polyethylene terephthalate (PET), polystyrene (PS), multilayer polyethylene terephthalate–ethylene-vinyl alcohol copolymer–polyethylene (PET–EVOH–PE), multilayer polyethylene–polyethylene terephthalate (PE–PET), polyvinyl chloride aluminum (PVC–AL), and polylactic acid (PLA), which were provided by manufacturing companies in the sector. PE, PP, and PLA activated with tyrosol, zinc oxide, or zinc acetate were also tested. The phenomena and properties, such as overall migration, thermal behavior, oxygen barrier, and physical properties were analyzed before and after the process. The results show that the HHP process only slightly affected the properties of the materials. After pressurization, oxygen permeability increased greatly in PVC–AL (from 7.69 to 51.90) and decreased in PLA (from 8.77 to 3.60). The additivation of the materials caused a change in color and an increase in oxygen permeability. The additivated PE and PP showed migration values above the legal limit for certain simulants. The HHP treatment did not greatly affect the mechanical properties of the additivated materials. The main increases in the migration after HHP treatment were observed for PE activated with tyrosol or zinc oxide and for PS activated with zinc oxide. Activated PLA performed the best in the migration studies, irrespective of the HHP treatment. The results suggest that activated PLA could be used in HHP food processing as an inner antimicrobial layer in contact with the food packed in a container with the desired oxygen permeability barrier.

## 1. Introduction

Historically, food preservation methods based on heat treatments have been the most widely used. However, the market demands preservation and packaging methods that do not affect the organoleptic properties or nutrients of the food, while at the same time decreasing the use of additives and preservatives. In this framework, high hydrostatic pressure (HHP) treatments gain relevance as a physical procedure that allows the inactivation of certain enzymes and microorganisms, while preventing or delaying food spoilage. This is because most of the decomposition reactions of nutrients, such as vitamins, pigments, and compounds that affect flavor, are not accelerated by HHP (a fact that does occur with an increase in temperature). HHP treatments are increasingly used as a final processing step in food preservation because, despite having a slightly higher cost than conventional heat treatments, the benefits make packaging companies opt for this preservation technique. Among the applications of HHP are the pasteurization of fruits and vegetables, meat tenderization, texturization of fish proteins, treatment of dairy products, and freezing/thawing of foods [1,2].

HHP treatments, like others, such as irradiation and ozone treatment, require that the food be packaged. The characteristics of the container will be decisive for the food preservation method to be effective and subsequently prevent environmental factors from affecting the food. The characteristics of a barrier element and its mechanical properties will determine which material is to be used as packaging. These characteristics, together with the low cost of the raw material, make plastic polymers an ideal material for food packaging. The treatment of packed foods by HHP implies that, in addition to affecting the characteristics of the food, the properties of the package may also be affected, and it may become ineffective as a barrier element. Therefore, the packaging materials should be selected so that they ensure and maintain its barrier properties, seal strength, transparency, and gloss, even after processing [3,4,5,6].

There are several studies reporting on the changes of the properties of materials, mostly plastics, but also nanomaterials, after being subjected to HHP treatments (reviewed by [4,6,7,8,9,10,11,12,13]). However, there are not many studies on how it affects global migration values, a very important parameter to consider in plastic materials intended to be in contact with food, as established by the European Regulation EU 10/2011 and its subsequent amendments, through which an overall migration limit of 10 mg/dm^2^ is established. Furthermore, plastic materials may be additivated with antimicrobial substances, providing an additional protection barrier against food spoilage bacteria and foodborne pathogens [14]. In this work, the plastic materials were additivated with substances of an antimicrobial nature, some authorized by the European Food Safety Authority (EFSA), such as zinc oxide and zinc acetate, and others not yet authorized, but which have positive studies by the EFSA (tyrosol). In a previous study, it was shown that plastic materials additivated with tyrosol or zinc derivatives showed antibacterial activity against food spoilage bacteria and/or foodborne pathogens [15]. This opens interesting possibilities for the application of the additivated materials in food preservation singly or in combination with other options, such as HHP treatments. However, the additivation process often changes the properties of the polymeric material obtained, which can influence its behavior after being treated by HHP. Therefore, it is of utmost importance to determine the possible changes in the additivated materials used in the present study after application of the HHP treatment. The aim of the present study was to determine the impact of the HHP treatment on the properties of the plastic materials polypropylene (PP), polyethylene (PE), and polylactic acid (PLA) activated or not activated with the antimicrobials tyrosol, zinc acetate, and zinc oxide. The parameters of the HHP treatment chosen for the study (680 MPa, 8 min) were slightly above those applied most frequently by the food industry (600 MPa, 4 min) in order to have a safety margin on the results. A second objective was to determine the impact of the HHP treatment on the physical and barrier properties of other plastic materials most commonly used in the food industry, such as polyethylene terephthalate (PET), polystyrene (PS), multilayer polyethylene terephthalate–ethylene-vinyl alcohol copolymer–polyethylene (PET–EVOH–PE), multilayer polyethylene–polyethylene terephthalate (PE–PET), and polyvinyl chloride aluminum (PVC–AL).

## 2. Materials and Methods

### 2.1. Materials

For the present research work, plastic polymeric materials used exclusively in the food industry were used, specifically those that come into direct contact with the food because they will serve as a container to house it, preserve it, extend its shelf life, and guarantee its quality and safety. The materials that were tested are the following: extruded polystyrene (PS) foil, multilayer polyethylene terephthalate–ethylene-vinyl alcohol copolymer–polyethylene (PET–EVOH–PE) foil, polyvinyl chloride aluminum (PVC–AL) foil, polypropylene (PP) foil, polyethylene (PE) foil, polyethylene terephthalate (PET) foil, polylactic acid (PLA) foil, and polyethylene–polyethylene terephthalate (PE–PET) bilayer foil. The plastic materials were obtained thanks to the courtesy of companies that manufacture plastic materials and containers for food use and with which the laboratory of the Andaltec Plastic Technology Center (Martos, Spain) maintains a close relationship. The materials PP, PE, and PLA were also tested in activated forms with 5% tyrosol (Sigma-Aldrich, Madrid, Spain), 10% or 1% zinc oxide (Sigma-Aldrich), or 5% or 0.5% zinc acetate (Sigma-Aldrich). The choice of these compounds was based on their high antibacterial activity, their acceptance or favorable studies by the EFSA/FDA as additives for use in food contact polymeric materials, their processability by extrusion and injection, and their price. For activation, the mastermixes containing the polymer and additives in the desired proportion were extruded through an SJH-20 extruder (Siepla Ingenieria, S.L., Barcelona, Spain) in order to obtain the desired films.

### 2.2. Scanning Electron Microscopy

Activated films were examined under a high-resolution scanning electron microscope (MERLIN model, Carl Zeiss, Jena, Germany) equipped with a nitrogen injection load compensation system.

### 2.3. High Hydrostatic Pressure Treatment

**Figure 1:**
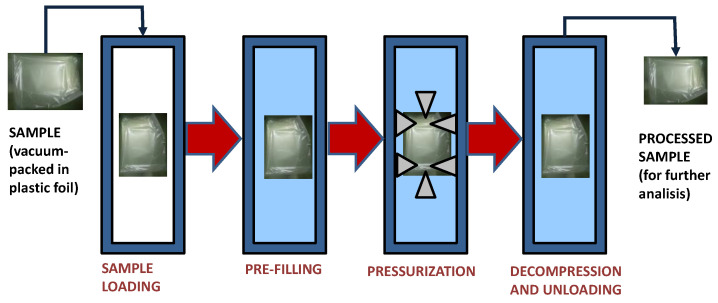
Operation scheme of the high hydrostatic pressure treatment.

High hydrostatic pressure (HHP) treatments were applied with a Stansted Fluid Power LTD HHP equipment (SFP, Essex, UK) suited with a 2.5 l vessel capable of operating in a pressure range of 0 to 700 MPa, under nonthermal conditions. Plastic samples were treated inside polyethylene–polyamide bags hermetically sealed (Figure 1). Samples were pressurized at 680 MPa for 8 min. These conditions were chosen as a slightly more intense treatment compared to the conditions applied most often by commercial systems (600 MPa, 4 to 5 min). The system was operated at a come-up speed of 75 MPa/min and almost immediate decompression. Pressurization fluid was water with added 10% propylenglycol. The temperature inside the vessel during treatments ranged between 23 and 27 °C.

### 2.4. Tensile Tests

To determine the mechanical properties of the materials, tensile tests were carried out according to the UNE-EN ISO 527-1 and UNE-EN ISO 527-3 standards. The equipment used for the tensile test was a Tinius Olsen Universal Testing Machine (Tinius Olsen TMC; Horsham, PA, USA) with 100 N and 10 KN load cells.

The following specimens were used, whose dimensions conform to the regulations of the tests carried out: Type 1BA traction specimens according to the UNE-EN ISO 527-3 standard. They were obtained by machining from the starting samples. In all cases, the test pieces were torsion-free and had mutually perpendicular parallel surfaces. Surfaces and edges were free of scratches, voids, nicks, and burrs. Compliance with these requirements was controlled by visual observation of the straightness of the edges, perpendicularity, and the smoothness and through the measurement with micrometric calipers. Specimens showing any observable or measurable lack of one or more of these requirements were removed or machined to the correct dimensions and shape prior to testing. All surfaces of the specimens were free of visible defects, scratches, or other imperfections. Once the specimens had been prepared for each type of test to be carried out, they were sized and stored in an oven at 40 °C in order to condition them to avoid any possible alteration.

### 2.5. Determination of Light Transmission

To determine the transmission of light, a X-Rite color i7 colorimeter (X-Rite Inc., Grand Rapids, MI, USA) operating in the range of 400 to 700 nm was used. To do this, the sample of dimensions determined according to specifications was introduced into the colorimeter, and the color transmission was measured before and after the HHP treatment.

### 2.6. Determination of Oxygen Transmission Rate

In the measurement of the degree of permeability to oxygen, a Labthink equipment (Labthink Instruments Co., Ltd., Jinan, China) with capacity for the determination of samples in foil or bottle format was used. The test was carried out under the provisions of ISO 15105-1:2007. The mechanized sample with a specific tool for this purpose in a spherical shape as specified in the test standard was introduced into the compartment intended for determining the passage of oxygen through.

### 2.7. Infrared Spectrometry

To identify any variation that the material may have suffered after the process in its chemical structure, infrared spectra were recorded with a Bruker infrared spectrophotometer (model Tensor 27; Bruker, Billerica, MA, USA). The equipment measures in the near-infrared range and has an ATR to be able to measure solid materials directly by placing them in the apparatus without the need for prior conditioning of the sample.

### 2.8. Differential Scanning Calorimetry

Differential scanning calorimetry (DSC) experiments were carried out in a Mettler Toledo DSC calorimeter (Mettler Toledo Inc., L’Hospitalet de Llobregat, Spain) in a temperature range of 30 to 320 °C with a heating rate of 10 °C/min in a nitrogen atmosphere of 50 mL/min.

### 2.9. Migration Tests

To determine the substances that migrate from the plastic material to the food in a container containing food, a global migration test was carried out under the UNE-EN ISO 1186: 2OO2 regulations in their corresponding sections. The Andaltec laboratory (Martos, Spain) is accredited by ENAC to carry out this test. The simulants and conditions for migration studies were selected according to European Regulation EU 10/2011, according to the intended use of the packaging material (including food type, prolonged storage, refrigeration, freezing, and heating in an oven or microwave). The food simulants established by said Regulation are: A (20% ethanol), B (3% acetic acid), C (20% ethanol), D1 (50% ethanol), and D2 (vegetable oil). Analyses were performed with a Bruker 430-GC gas chromatographer. Due to the versatility of the plastic materials that were used in the study, the conditions of this test were different for each one. PET (B), PS (D1), PET-EVOH-PE (A), PE-PET (D2) and PVC-AL (D2) were incubated with simulants (in parenthesis) for 2 h at 70 °C. The rest of materials were incubated with simulants A, B and D2 for 10 days at 40 °C. 

## 3. Results

### 3.1. Electron Microscopy of Activated Films

**Figure 2:**
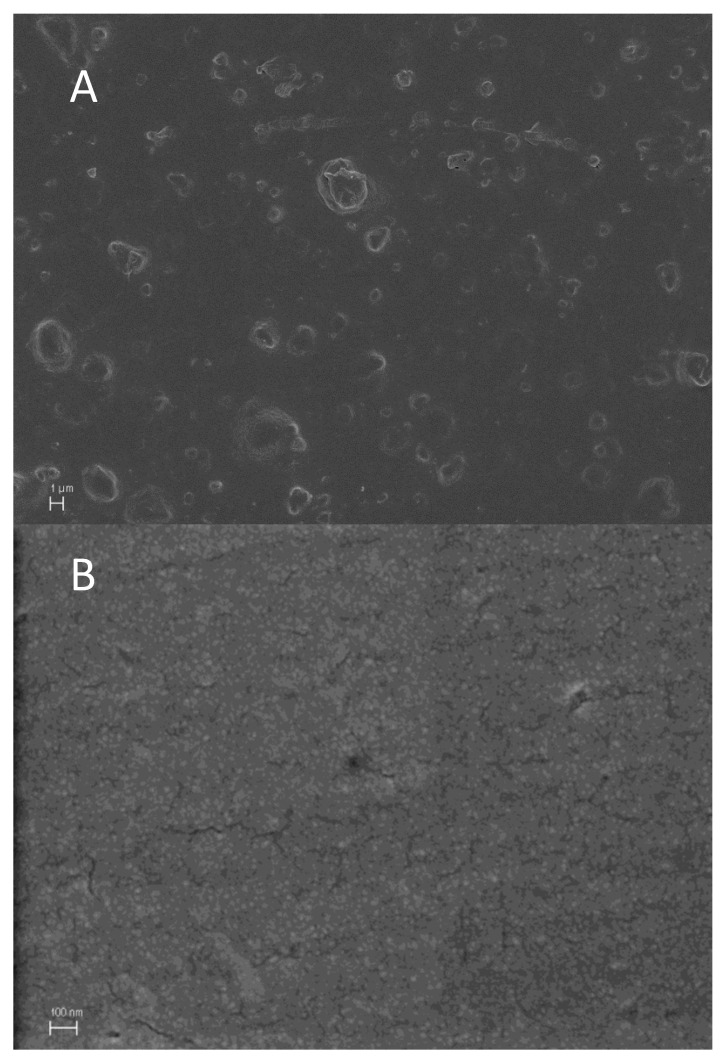
Scanning electron microscopy of PP activated with 5% zinc acetate (**A**) and PE activated with 5% tyrosol (**B**).

Activated films were characterized by scanning electron microscopy to observe the distribution of the additives in the plastic matrix (Figure 2). The most representative images are shown below, where a greater homogeneity and uniformity can be appreciated in the tyrosol-additivated material, contrary to what happens with zinc acetate, which presents aggregates.

### 3.2. Tensile Tests

The results of the tensile tests of the plastic materials before and after being treated by HHP are shown in Table 1.

After HHP treatment, PS showed a slight decrease both in the load at the breaking point and in the elongation that occurs in the material until it breaks.

In PET–EVOH–PE, all the parameters decreased considerably. The material became less resistant to elongation after being subjected to high-pressure treatment.

For PVC–AL, its tensile properties increased slightly after treatment. It increased the load they support before breaking as well as the elongation that occurs.

In PET, the load supported before breaking increased slightly; however, the pressurized material was less elongated; that is, after treatment, the material loses elasticity.

In PLA, all the parameters were slightly higher after treatment, but it should be noted that the load it supports before the breaking point increased and it became somewhat more elastic as it lengthened somewhat more.

In PE–PET, the maximum load it supports slightly decreased until the material broke, and the elongation that occurred up to the breaking point slightly increased.

In the pressurized PE, the maximum load increased, while elongation decreased. The addition of 5% tyrosol (PE–Tyr 5%) decreased the maximum supported load and the elongation of the material compared to PE. The supported load still decreased further when PE–Tyr 5% was pressurized, although its elongation improved. The addition of 10% zinc oxide (PE–ZnO 10%) did not modify the load supported but it decreased elongation. When the material was pressurized, the load supported decreased, but elongation increased considerably.

**Figure 3:**
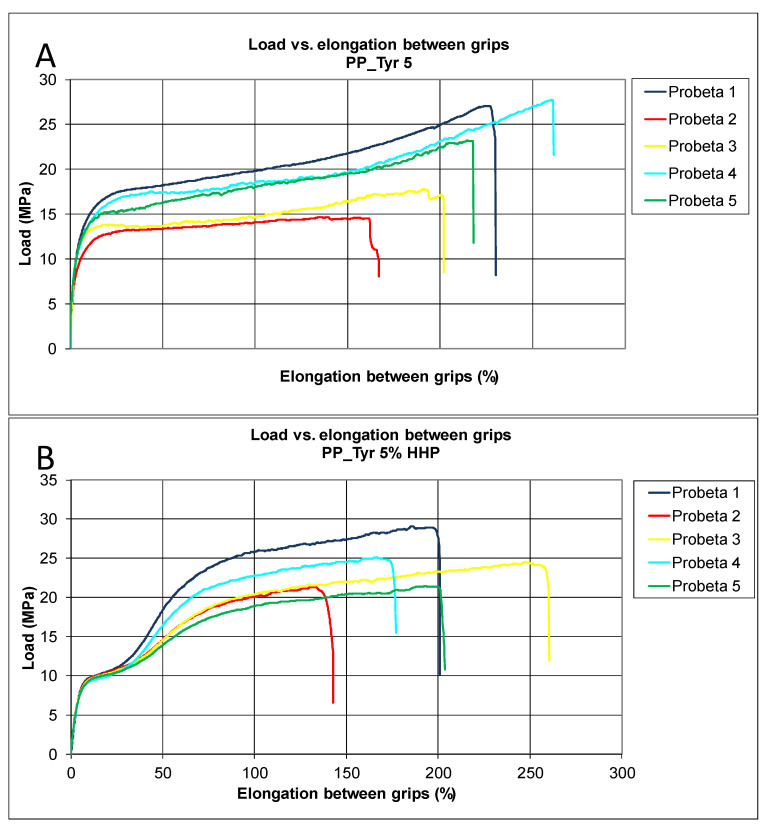
Results of tensile tests on PP–Tyr 5% before (**A**) and after HHP treatment (**B**).

In PP, the HHP treatment caused a decrease in the load supported and a slight decrease in elongation (Figure 3). The addition of 5% tyrosol to the material (PP–Tyr 5%) did not modify the load supported, but it did decrease the elongation value considerably compared to PP. The HHP treatment of PP–Tyr 5% did not modify considerably the load supported or the elongation. The incorporation of zinc oxide in the material (PP–ZnO 10%) decreased the load supported before breaking very considerably, and it also decreased elongation. The pressurization of the material seemed to improve its supported load, but it decreased elongation. When PP was modified by adding 5% zinc acetate (PP–ZnAc 5%), the supported load decreased slightly, but elongation decreased more considerably. The pressurization of PP–ZnAc 5% resulted in considerably higher values for the supported load and elongation.

In PLA, the HHP treatment increased both the supported load and elongation. The modification of the material by the addition of 5% tyrosol (PLA–Tyr 5%) improved the supported load but did not affect elongation. After pressurization, the supported load and elongation of PLA–Tyr 5% decreased, but the value of the supported load was still higher compared to PLA without any additive. The addition of 1% zinc oxide to PLA (PLA–ZnO 1%) improved the supported load considerably and decreased its elongation compared to PLA. The HHP treatment had almost no effect on the parameters of PLA–ZnO 1%. The modification of PLA with 0.5% zinc acetate (PLA–ZnAc 0.5%) also improved the supported load and elongation compared to PLA. The pressurization of PLA–ZnAc 0.5% did not modify the studied parameters of the material.

### 3.3. Colorimetry Test

In the materials tested before and after being subjected to the HHP treatment, the resulting color spectra were practically identical, except for PLA, in which there was a slight decrease in the percentage of light transmission after the HHP treatment (Table 2). The lack of changes in light transmission suggested that they were not affected by HHP processing. The mix with additives did result in a considerable loss of light transmission that was proportional to the concentration of the additive. However, PP and PE films containing additives did not show remarkable changes in light transmission after pressurization. By contrast, the decreases in the percentages of light transmission were observed in the pressurized PLA–Tyr 5%, PLA–ZnAc 0.5%, and PLA–ZnOx 1%.

### 3.4. IR Spectroscopy Test

**Figure 4:**
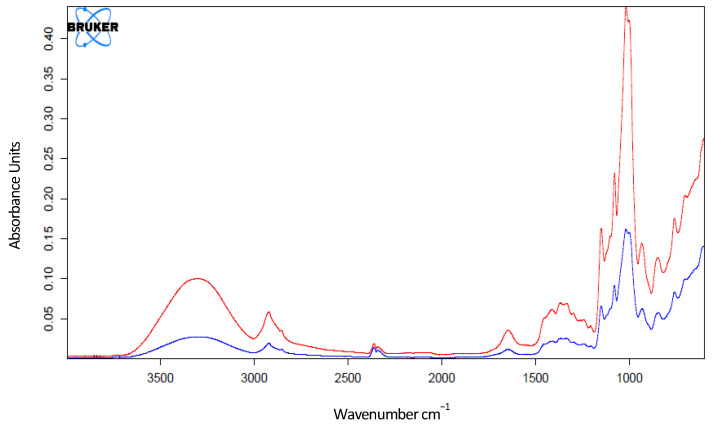
Decrease in peak intensity of PLA after treatment by HHP (blue line) compared to the untreated control (red line).

From the comparison of the spectra resulting from the materials before and after the application of the HHP treatment, no considerable differences were detected, suggesting that there was no degradation or change in the different materials. The main changes detected after the application of HHP involved an increase in peak intensity for PET–EVOH–PE, PVC–AL, PE–Tyr 5%, and PLA–ZnAc 0.5%. Similarly, a decrease in peak intensity was observed for the following materials after application of the HHP treatment: PET, PLA (Figure 4), and PLA–ZnOx 1%.

### 3.5. DSC

**Figure 5:**
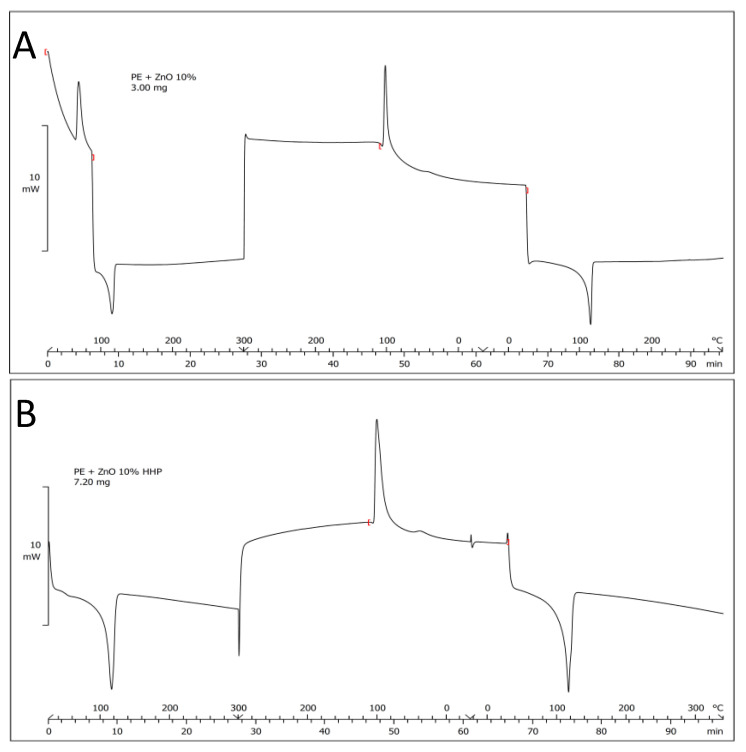
DSC PE–zinc oxide 10% before (**A**) and after HHP treatment (**B**).

The results from the DSC tests suggest that the application of the HHP treatment did not influence the different plastic materials used in the study. DSC was performed on additives before they were incorporated into the plastic materials for comparison (Figure 5). Tyrosol showed an endothermal peak at 94 °C corresponding to its melting. It also showed changes in the spectrum above 170 °C related to the degradation of the compound. Zink oxide showed an endothermal peak at 250 °C, which was attributed to changes in its crystalline structure. Zinc acetate showed several endothermal peaks, at temperatures of 100 °C (related to water loss of the hydrated form) and 250 °C (degradation of the molecule with a loss of CO_2_, acetone, and acetic acid). The DSC signals were much less pronounced when the tests were carried out with the compounds incorporated into the plastic materials PPE, PP, and PLA. Furthermore, no detectable changes were observed in the DSC spectra when the activated plastic materials were treated by HHP.

### 3.6. Oxygen Permeability

The oxygen transmission rates (OTRs) of the different materials before and after HHP treatment are shown in Table 3. PS was the material with the highest OTR, and it showed an additional increase in the OTR after being treated by HHP. PET–EVOH–PE is a high-barrier material, which translates into a very low permeability to oxygen. The values obtained before and after treatment are similar and offer little variation. For PVC–AL, there was a considerable increase in the permeability after HHP treatment. PET did not show a high permeability, but a considerable increase was still observed after pressurization. The PE–PET material has very low permeability, with little change after the HHP treatment. For PLA, unlike in other materials, a decrease in the permeability value was obtained in the treated material.

The rest of the materials included in the study for activation with tyrosol, zinc oxide, or zinc acetate showed high increases in oxygen permeability in the preliminary tests following incorporation of the additives and therefore were not included in the study.

### 3.7. Migration

To carry out the migration tests, the use to which each material is usually destined was studied a priori, that is, the foods with which it is expected to come into contact fundamentally. Based on this study, the test conditions were established as well as the type of food simulant in which it has contacted. Table 4 shows the results obtained from all the global migration tests according to the UNE-EN 1186 standard on all materials before and after the HHP treatment.

PET is used mainly for packaging and is in contact with dairy desserts, sandwiches, water, soft drinks, and juices, and thus food simulant B seemed the most adequate. After the HHP treatment, slightly higher migration values were obtained, but they were still below the established legal limit of 10 mg/dm^2^.

PS is usually used to pack dairy desserts, desserts, and ice cream. In this case, the D1 simulant was used. The results of the global migration did not show differences between the controls and pressurized samples. Furthermore, the values obtained were very low and far from the established limit.

Due to its barrier properties, PET–EVOH–PE is used for many purposes in food packaging, such as being in contact with meat products, fresh pasta, salads, cheeses, and sausages. Simulant A, which is common to all of them, was used in the tests. The results obtained were similar for the controls and pressurized samples, and the migration values were very low and far from exceeding the established legal limit.

PE–PET is often used for the packaging of cheeses, sausages, pizzas, fresh pasta, meats, nuts, etc. The simulant D2 was used. In this case, the migration values obtained after the HHP treatment were slightly higher compared to the controls, but still far below legal limits.

In the case of PVC–AL, it is important to study migration, since the possible migration of phthalates from PVE into food is a very worrying issue. After testing PVC–AL with the simulant D2, the results obtained after application of the HHP treatment were only slightly higher, and in both cases (controls and pressurized samples), the values were low and far from the established legal limit.

PE pressurization resulted in only small increases in the migration of simulants. However, when PE was activated with 5% tyrosol (PE–Tyr 5%), the migration of simulants increased considerably and above the established limits. The application of HHP treatment to (PE–Tyr 5%) only induced small increases in the migration of the simulants. In the case of PE activated with 10% zinc oxide (PE–ZnO 10%), the migration of simulant B increased considerably above legal limits. However, the HHP treatment of PE–ZnO 10% only caused a small increase in the migration of simulant B.

PP pressurization did not increase the migration of simulants. The activation of PP with 5% tyrosol (PP–Tyr 5%) increased the migration of simulant D2 above legal limits, but did not affect the migration of simulants A or B. The HHP treatment only caused a small increase in the migration of simulant D2 in PP–Tyr 5%. The activation of PP with 10% ZnO (PP–ZnO 10%) increased the migration of simulant B above legal limits, while the migration of simulants A and D2 was not affected. The pressurization of PP–ZnO 10% only increased the migration of simulant B slightly. By contrast, the activation of PP with 5% zinc acetate did not increase the migration of any of the simulants tested, and the same was observed when PP–ZnAc 5% was treated by HHP.

The application of the HHP treatment to PLA did not increase the migration of any of the simulants tested. The PLA activated with 5% tyrosol (PLA–Tyr 5%) showed an increased migration of simulant D2, which was below legal limits. The migration of simulant D2 increased slightly when PLA–Tyr 5% was pressurized, but the migration value was still below legal limits. PLA activated with 1% zinc oxide or with 0.5% zinc acetate did not show changes in the migration of simulants regardless of the application of HHP treatment.

## 4. Discussion

The results from the present study indicate that, in the colorimetry test, most of the materials maintained their color transmission values before and after HHP treatment; i.e., their degree of transparency remained practically unchanged after treatment. However, the materials activated with the antimicrobials tyrosol, zinc oxide, and zinc acetate lost the initial transparency of the base plastic material, as in the cases of PE, PP, and PLA polymers. Furthermore, the electron microscopy examination showed that the degree of dispersion of these matrices was not completely homogeneous, because they generate some areas of agglomerate accumulation in the plastic matrix, especially in the case of zinc oxide.

For the materials activated with antimicrobial agents, it was observed that, in all cases, the samples obtained both before and after undergoing the HHP treatment (680 MPa for 8 min) showed lower light transmission percentage values than those of the base material (without additives). Remarkably, PLA as well as activated PLA forms showed a slight reduction in light transmission after pressurization. Previous studies have reported an increased opacity of films after HHP treatments, an effect that has been attributed to a possible closure of spaces in the polymeric chemical structure due to the applied pressure [16]. By contrast, another study reported no significant change in the light barrier properties of multilayer films, such as LDPE/PA/LDPE, LDPE/EVOH/LDPE, PET/LDPE/PA/EVOH/PA/LDPE, and PET/Al/PA/PP [17]. A small change in the light transmission would only affect the visual aspect of the packaging, but would not influence the food preservation. The opposite (an increase in transmission) would be detrimental to the food, accelerating food deterioration phenomena due to light exposure.

In most cases, the color and degree of transparency of a container are determining factors in keeping the qualities of the foodstuff it contains practically unaltered. Depending on the food it contains, it is desirable that the transparency is maintained or not throughout its shelf life. Thus, for example, for foods that are negatively affected by the effect of light, it will be advisable for the packaging to be dark or opaque, and for those that are not affected by light, the greater the transparency the better, as the better the perception of the food it contains; we must also bear in mind that the consumer’s opinion of the food is very important, as its presentation plays a very important role in the final decision to buy.

From the results obtained in the IR spectra of each of the materials tested, we found that there is practically no variation in the spectra obtained for all the plastic materials before and after being subjected to the HHP treatment. This suggests that the applied pressure does not alter the molecular structure of the material and there is no degradation of the compound, so the macromolecule is not being broken down into other smaller ones that could be harmful to human health. These results are also in agreement with those obtained by DSC. Previous studies also indicated that HHP treatments had low or no effects on the thermal properties of plastic materials [18,19].

A very important factor in the use of plastic materials for food packaging is oxygen permeability. Foods that are affected by the presence of oxygen must be packaged in high-barrier packaging and, in most cases, in the presence of a modified atmosphere. Otherwise, negative effects, such as rancidity or growth of mold, may occur, thus considerably reducing the shelf life of the food. Since preliminary tests indicated that the polymers PE, PP, and PLA activated with tyrosol, zinc oxide, and zinc acetate had a high oxygen permeability, detailed permeability tests were ruled out. A high oxygen permeability would be a limiting factor for use of these materials with most foodstuffs. However, they still could be used as antimicrobial films in contact with the food packed in a second container of low oxygen permeability. Previous studies have shown that the incorporation of additives into polymeric materials may either increase or decrease oxygen permeability, depending on the material and the additive. For example, a recent study indicated that the incorporation of plant extracts into chitosan films resulted in a reduction in oxygen permeability [20], an effect that could be explained by the possible crosslinking between components of the extracts and the polymer matrix [21].

Regarding the other materials tested, we observed low increases in oxygen permeability after HHP treatment, except for PVC–AL, where there was a considerable, unstable, and progressive increase, and in the case of PLA, where the permeability value decreased. This could be explained by assuming that the gaps between the polymer molecules are reduced or closed during HHP treatment. Previous studies reported that the permeability of plastic materials was not affected by high-pressure treatments at pressures from 200 to 600 MPa [22] or between 200 and 550 MPa [23]. However, in other cases, a loss of barrier properties was found due to the decompression of the polymer molecules caused by the treatment [24]. The reverse effect can also be observed in some studies; i.e., treatment by high pressures caused the molecules to compact, thus improving the barrier properties of the packaging material [6,25].

As for the tensile strength results, after the high-pressure treatment was applied, the values obtained showed in general a decrease in the load supported until the material broke and loss of elasticity, except in the cases of PE–PET, in which there was a decrease in the load supported and an increase in the elongation, PVC–AL, and PLA, in which all the parameters increased slightly. Previous studies have described that high-pressure treatments can lead to changes in the mechanical properties of the packaging, such as delamination [6]. Changes in mechanical properties can also affect the packaging integrity, barrier properties, and mechanical strength. In addition, changes in mechanical properties it can also affect the strength after heat sealing, which is highly dependent on the properties of the inner layer of the packaging material and is essential to maintain the integrity of the package and preserve the quality of the packaged product. Other studies, however, reported that HHP in the range of 200 to 600 MPa for 10 min at 10 °C minimally affects the mechanical strength of packaging materials [22].

According to the results obtained in this study, in all the materials without additives, the migration of volatile substances did not change or slightly increased after the high-pressure treatment. The migration values were very low and far from the established legal limits and therefore do not pose any danger to human health. However, in the case of additivated materials, the migration values in certain food simulants exceed the migration limits of 10 mg/dm² established by European Regulation EU 10/2011. Migration is one of the most important aspects to consider when packaging materials are subjected to high-pressure treatment. Substances migrating from packaging to products can affect the sensory quality and toxicity level of the packaged product. Specifically, food and packaging interact through mass transfer mechanisms, such as permeation, sorption, and migration [4,6].

The results obtained in previous migration studies on nonadditivated packaging materials are largely in agreement with those obtained in the present study. For example, in the work by [26], the total migration of laminated PA/LDPE and coextruded PA/LDPE materials were processed at 400 and 500 MPa, 20 °C/15 min, and the migration values for different simulants were not modified after processing, showing that these materials are suitable for use in food contact subject to the conditions of that study. Galotto et al. [27] evaluated four packaging materials (LDPE/EVOH/LDPE, PETmet/LDPE, PET/LDPE, and PPSiOx) after HHP pressure processing at 400 MPa/20 and 60 °C/30 min. The total migration of all containers in contact with the aqueous simulant was <10 mg/dm^2^. However, compared to the unprocessed films, the total migration was significantly lower than that of the samples after the application of high pressure. The authors attributed these results to the fact that HHP processing causes a higher compaction of the polymer structure and, consequently, a higher degree of crystallinity. This phenomenon leads to a reduction in migration levels, as more organized structures limit the movement of compounds with migration potential. Furthermore, according to [28] and [29], during high-pressure compression, the polymer matrix loses its ability to release components from the packaging due to the reduction of free volume. As the pressure is released, the polymer quickly regains its original dimensions, and, therefore, migration processes continue as expected at normal atmospheric pressure. Therefore, migration values can be statistically equal to samples of containers that were not subjected to high-pressure processing.

## 5. Conclusions

From the analysis of the results obtained, we can conclude that the HHP treatment tested does not alter the molecular chemical structure of the materials studied. It does not increase or only slightly increases migration in the unadditivated packaging materials studied. The additivation of PE and PP with tyrosol or zinc oxide increases the migration values for certain food simulants above the established migration limits. The HHP treatment does not cause changes in light transmission in the unadditivated materials, while the additivated materials show lower transmission rates. Oxygen permeability increases only slightly after high-pressure treatment for most of the materials, except for PVC–AL, where there is a considerable, unstable, and progressive increase, and PLA, where the permeability value decreases. Most of the samples treated by HHP show a slight decrease in the load supported until the material breaks and a loss of elasticity, except in the cases of PE–PET, where the load supported decreases and the elongation due to traction increases, and the PVC–AL and PLA materials, where all the parameters increase slightly. Therefore, it can be concluded that the HHP treatment tested does not have a considerable and relevant effect on the plastic materials used in the food packaging, so that most of them are suitable to be used for this purpose. The results also indicate that additivated PE and PP are the most prone to changes in parameters, such as migration, oxygen permeability, and light transmission, while PLA seems to be the most suitable for additivation.

## Figures and Tables

**Table 1:** Results of tensile tests on the different materials before and after being treated by HHP.

	Load Supported	Elongation at Break	Young’s Module
Material	σB (MPa)	εB (%)	(MPa)
PS	14.80 ± 1.70	8.80 ± 4.20	2090.00 ± 114.00
PS (HHP)	13.40 ± 4.30	6.30 ± 4.90	2140.00 ± 55.00
PET–EVOH–PE	29.02 ± 15.00	240.00 ± 130.00	1550.00 ± 779.00
PET–EVOH–PE (HHP)	8.20 ± 0.97	8.80 ± 4.20	1159.00 ± 63.30
PVC–AL	21.62 ± 7.90	31.20 ± 8.10	2280.00 ± 220.00
PVC–AL (HHP)	28.20 ± 4.73	52.00 ± 23.03	2434.00 ± 48.00
PET	23.10 ± 6.50	7.50 ± 5.80	1610.00 ± 522.00
PET (HHP)	32.50 ± 5.90	4.32 ± 0.28	1830.00 ± 256.00
PLA	13.03 ± 5.60	9.15 ± 0.80	1501.00 ± 94.40
PLA (HHP)	18.20 ± 5.50	11.10 ± 2.10	1840.00 ± 584.00
PE–PET	35.80 ± 7.30	205.00 ± 95.01	620.00 ± 173.00
PE–PET (HHP)	31.40 ± 6.60	240.02 ± 19.03	1090.00 ± 621.00
PE	22.75 ± 3.50	195.14 ± 43.47	196.46 ± 10.71
PE (HHP)	27.31 ± 1.13	109.45 ± 16.33	147.39 ± 12.13
PE–Tyr 5%	19.31 ± 2.07	121.65 ± 22.00	140.47 ± 14.43
PE–Tyr 5% (HHP)	17.62 ± 0.92	155.17 ± 34.18	143.83 ± 11.07
PE–ZnO 10%	22.00 ± 3.62	145.34 ± 22.06	231.35 ± 15.85
PE–ZnO 10% (HHP)	15.82 ± 3.69	387.20 ± 6.76	370.06 ± 65.32
PP	22.11 ± 3.41	463.67 ± 73.43	460.82 ± 64.24
PP (HHP)	17.24 ± 1.81	445.13 ± 70.49	361.62 ± 38.82
PP–Tyr 5%	21.83 ± 5.64	212.53 ± 37.27	336.09 ± 44.07
PP–Tyr 5% (HHP)	22.75 ± 3.50	195.14 ± 43.47	196.46 ± 10.71
PP–ZnO 10%	8.78 ± 2.04	222.88 ± 75.69	91.26 ± 18.44
PP–ZnO 10% (HHP)	17.10 ± 3.70	129.50 ± 26.10	146.38 ± 38.18
PP–ZnAc 5%	17.31 ± 5.53	285.28 ± 26.01	307.85 ± 39.21
PP–ZnAc 5% (HHP)	29.75 ± 1.98	585.31 ± 35.76	_
PLA	13.01 ± 5.60	9.15 ± 0.80	1501.01 ± 94.40
PLA (HHP)	18.20 ± 5.50	11.10 ± 2.10	1840.03 ± 584.01
PLA–Tyr 5%	39.80 ± 3.03	8.96 ± 1.60	1284.85 ± 249.81
PLA–Tyr 5% (HHP)	15.80 ± 4.40	1.35 ± 0.37	1239.65 ± 232.92
PLA–ZnO 1%	27.30 ± 5.64	2.25 ± 0.39	1526.97 ± 286.23
PLA–ZnO 1% (HHP)	26.15 ± 10.83	1.89 ±0.70	1575.38 ± 745.68
PLA–ZnAc 0.5%	22.70 ± 7.10	1.90 ± 0.50	1256.78 ± 534.84
PLA–ZnAc 0.5% (HHP)	22.33 ± 11.19	1.90 ± 0.83	1236.69 ± 431.17

**Table 2:** Results of colorimetric tests of materials before and after being treated by HHP.

Material	Transmission (% T)	Color (CIE Scale: Lab D65-10)
PS	0	0_neutral
PS (HHP)	0	0_neutral
PET–EVOH–PE	15	40_yellow
PET–EVOH–PE (HHP)	15	40_yellow
PVC–AL	0	0_neutral
PVC–AL (HHP)	0	0_neutral
PET	100	100_yellow
PET (HHP)	100	100_yellow
PE–PET	15	40_yellow
PE–PET (HHP)	15	40_yellow
PE	70–80	0_neutral
PE (HHP)	70–80	0_neutral
PE–Tyr 5%	45–55	0_neutral
PE–Tyr 5% (HHP)	45–55	0_neutral
PE–ZnO 10%	0–22	20_yellow
PE–ZnO 10% (HHP)	0–22	20_yellow
PP	55–60	0_neutral
PP (HHP)	50–55	0_neutral
PP–Tyr 5%	5–30	20_yellow
PP–Tyr 5% (HHP)	5–30	20_yellow
PP–ZnO 10%	40–50	0_neutral
PP–ZnO 10% (HHP)	40–50	0_neutral
PP–ZnAc 5%	30–40	0_neutral
PP–ZnAc 5% (HHP)	30–40	0_neutral
PLA	85	0_neutral
PLA (HHP)	75–80	0_neutral
PLA–Tyr 5%	80	0_neutral
PLA–Tyr 5% (HHP)	70	0_neutral
PLA–ZnO 1%	65–85	0_neutral
PLA–ZnO 1% (HHP)	55–75	0_neutral
PLA–ZnAc 0.5%	80–85	0_neutral
PLA–ZnAc 0.5% (HHP)	80–85	0_neutral

**Table 3:** Results obtained for oxygen permeability tests.

Material	OTR ^1^ (cm³/m²/24 h/atm)
PS	459.00
PS (HHP)	500.00
PET–EVOH–PE	2.03
PET–EVOH–PE (HHP)	1.58
PVC–AL	7.69
PVC–AL (HHP)	51.9
PET	2.52
PET (HHP)	6.84
PE–PET	3.00
PE–PET (HHP)	3.58
PLA	8.77
PLA–HHP (HHP)	3.60

^1^ OTR, oxygen transmission rate.

**Table 4:** Overall migration test results for materials and food simulants, expressed in mg/dm². The legal limit according to EU Regulation 10/2011 for plastic materials in contact with food is 10 mg/dm².

Material	Migration Values (mg/dm²) for Food Simulants			
	A	B	D1	D2
PET ^1^		0.9 ± 0.4		
PET (HHP) ^1^		1.6 ± 0.4		
PS ^1^			1.1 ± 0.4	
PS (HHP) ^1^			1.1 ± 0.4	
PET–EVOH–PE ^1^	1.1 ± 0.4			
PET–EVOH–PE (HHP) ^1^	1.2 ± 0.7			
PE–PET ^1^				2.1 ± 1.0
PE–PET (HHP)^1^				3.1 ± 1.1
PVC–AL ^1^				3.1 ± 0.45
PVC–AL (HHP) ^1^				3.6 ± 0.65
PE ^2^	<4 ± 1.7	<4 ± 1.3		<4.4 ± 1.7
PE (HHP) ^2^	<4 ± 1.7	<4 ± 1.3		<4.4 ± 1.7
PE–Tyr 5% ^2^	10.5 ± 1.7	169.6 ± 20		21.8 ± 5.5
PE–Tyr 5% (HHP) ^2^	13.5 ± 1.7	175.2 ± 20		22.3 ± 5.5
PE–ZnO 10% ^2^	<4 ± 1.7	36.7 ± 4.0		<4.4 ± 1.7
PE–ZnO 10% (HHP) ^2^	<4 ± 1.7	38.3 ± 4.2		<4.4 ± 1.7
PP ^2^	<4 ± 1.7	<4 ± 1.3		<4.4 ± 1.7
PP (HHP) ^2^	<4 ± 1.7	<4 ± 1.3		<4.4 ± 1.7
PP–Tyr 5% ^2^	<4 ± 1.7	<4 ± 1.3		18 ± 4.5
PP–Tyr 5% (HHP) ^2^	<4 ± 1.7	<4 ± 1.3		19.3 ± 4.5
PP–ZnO 10% ^2^	<4 ± 1.7	19 ± 2.0		<4.4 ± 1.7
PP–ZnO 10% (HHP) ^2^	<4 ± 1.7	21.2 ± 2.0		<4.4 ± 1.7
PP–ZnAc 5% ^2^	<4 ± 1.7	<4 ± 1.3		<4.4 ± 1.7
PP–ZnAc 5% (HHP) ^2^	<4 ± 1.7	<4 ± 1.3		<4.4 ± 1.7
PLA ^2^	<4 ± 1.7	<4 ± 1.3		<4.4 ± 1.7
PLA (HHP) ^2^	<4 ± 1.7	<4 ± 1.3		<4.4 ± 1.7
PLA–Tyr 5% ^2^	<4 ± 1.7	<4 ± 1.3		5.8 ± 1.7
PLA–Tyr 5% (HHP) ^2^	<4 ± 1.7	<4 ± 1.3		7.4 ± 1.9
PLA–ZnO 1% ^2^	<4 ± 1.7	<4 ± 1.3		<4.4 ± 1.7
PLA–ZnO 1% (HHP) ^2^	<4 ± 1.7	<4 ± 1.3		<4.4 ± 1.7
PLA–ZnAc 0.5% ^2^	<4 ± 1.7	<4 ± 1.3		<4.4 ± 1.7
PLA–ZnAc 0.5% (HHP) ^2^	<4 ± 1.7	<4 ± 1.3		<4.4 ± 1.7

^1^ Migration was determined after incubation of the material with simulant for 2 h at 70 °C. ^2^ Migration was determined after incubation of the material with simulants for 10 days at 40 °C.
